# Sequence variant at 4q25 near *PITX2* associates with appendicitis

**DOI:** 10.1038/s41598-017-03353-0

**Published:** 2017-06-08

**Authors:** Ragnar P. Kristjansson, Stefania Benonisdottir, Asmundur Oddsson, Tessel E. Galesloot, Gudmar Thorleifsson, Katja K. Aben, Olafur B. Davidsson, Stefan Jonsson, Gudny A. Arnadottir, Brynjar O. Jensson, G. Bragi Walters, Jon K. Sigurdsson, Snaevar Sigurdsson, Hilma Holm, David O. Arnar, Gudmundur Thorgeirsson, Kristin Alexiusdottir, Ingileif Jonsdottir, Unnur Thorsteinsdottir, Lambertus A. Kiemeney, Thorvaldur Jonsson, Daniel F. Gudbjartsson, Thorunn Rafnar, Patrick Sulem, Kari Stefansson

**Affiliations:** 1 deCODE genetics/Amgen, Inc, Reykjavik, 101 Iceland; 2 Radboud University Medical Center, Radboud Institute for Health Sciences, Department for Health Evidence, PO Box 9101, 6500 HB Nijmegen, The Netherlands; 3 Netherlands Comprehensive Cancer Organisation, PO Box 19079, 3501 DB Utrecht, The Netherlands; 4 Department of Medicine, Landspítali – The National University Hospital of Iceland, Hringbraut, 101, Reykjavik, Iceland; 5 Faculty of Medicine, University of Iceland, Reykjavik, Iceland; 6 Department of Surgery, Landspítali – The National University Hospital of Iceland, Hringbraut, 101, Reykjavik, Iceland; 7 School of Engineering and Natural Sciences, University of Iceland, Reykjavik, Iceland

## Abstract

Appendicitis is one of the most common conditions requiring acute surgery and can pose a threat to the lives of affected individuals. We performed a genome-wide association study of appendicitis in 7,276 Icelandic and 1,139 Dutch cases and large groups of controls. In a combined analysis of the Icelandic and Dutch data, we detected a single signal represented by an intergenic variant rs2129979 [G] close to the gene *PITX2* associating with increased risk of appendicitis (OR = 1.15, P = 1.8 × 10^−11^). We only observe the association in patients diagnosed in adulthood. The marker is close to, but distinct from, a set of markers reported to associate with atrial fibrillation, which have been linked to *PITX2*. *PITX2* has been implicated in determination of right-left symmetry during development. Anomalies in organ arrangement have been linked to increased prevalence of gastrointestinal and intra-abdominal complications, which may explain the effect of rs2129979 on appendicitis risk.

## Introduction

Appendicitis is a condition whereby the inner lining of the appendix becomes inflamed. Appendicitis has a relatively high prevalence; 8.6% of all males and 6.7% of all females will at some point develop appendicitis^1^. Rate of diagnosis peaks in childhood, although around half of cases are diagnosed in adulthood (Supplementary Table 1). The median age of diagnosis for all cases in the US is 21^1^. Appendix perforation presents a significant risk in acute appendicitis, causing release of bacteria into the abdominal cavity and significantly worsening prognosis^2^. Obstruction of the appendiceal lumen appears to play an important role in the pathogenesis of appendicitis^3^, but the condition remains poorly understood^4^.

No sequence variants influencing risk of appendicitis have been reported. A family history of appendicitis greatly increases an individual’s risk, and twin studies suggest that genetics account for around 30% of appendicitis risk, with a strong sex-linked effect^5, 6^. We estimated the risk ratio among siblings (λ_S_) of Icelandic patients with appendicitis (N = 8,160) to be 1.95 by cross-matching with the nationwide genealogy^7^ (Supplementary Table 2).

In order to discover sequence variants associating with risk of appendicitis, we performed a genome-wide association study (GWAS) of appendicitis, combining data from Icelandic and Dutch populations.

## Results

We performed a GWAS on combined data from 7,267 Icelandic and 1,139 Dutch cases of appendicitis, and 327,134 Icelandic and 4,587 Dutch controls. The Icelandic cases were identified from hospital records indicating diseases of the appendix coded according to the International Classification of Diseases (ICD) between 1983 and 2015 (Supplementary Table 3), whereas the Dutch cases were self-reported. In Iceland, we test 32.5 million imputed markers identified through whole-genome sequencing of 15,220 Icelanders and subsequently imputed into chip-typed individuals through long-range haplotype phasing. Genotype probabilities were calculated for first and second-degree relatives of chip-typed individuals^8^. In the Netherlands we test 15,268,903 markers identified through whole-genome sequencing of 249 Dutch trios (GoNL). We combined the results of 11,290,636 markers found in both populations. When testing for association, we used weighted genome-wide significance thresholds depending on variant class^9^.

### A sequence variant at 4q25 associates with appendicitis

**Figure 1:**
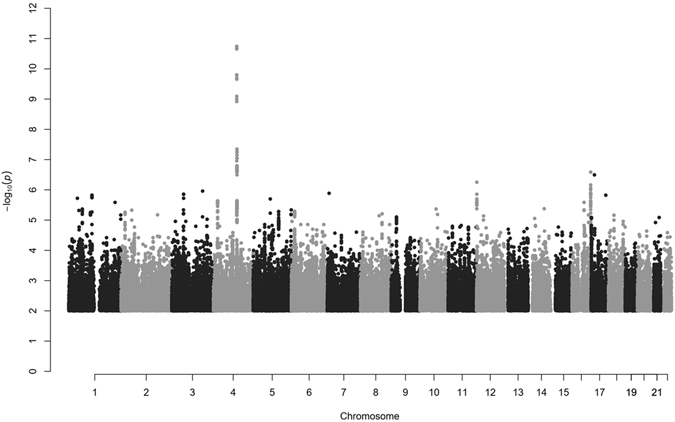
Manhattan plot for the combined Icelandic and Dutch appendicitis GWAS (N = 8,566). Only one region, 4q25, harbors a genome-wide significant signal. Variants are plotted by chromosomal position (x-axis) and -log_10_P values (y-axis). Individual Manhattan plots for the Icelandic and Dutch groups are shown in Supplementary Figures 4 and 5, respectively. Variants with P > 0.05 have been omitted.

**Figure 2:**
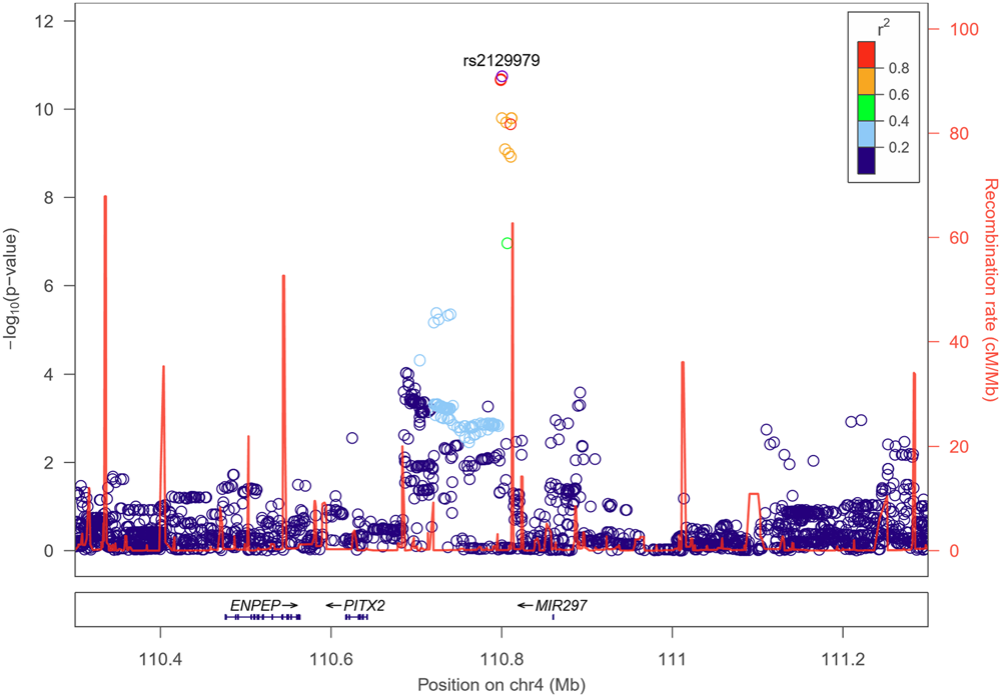
Locus plot for combined Icelandic and Dutch data showing the association of rs2129979 and surrounding variants at 4q25 with appendicitis (N = 8,566). The leading variant is shown as a purple circle, and other variants are coloured according to correlation (r^2^) with the leading marker (legend at top-right). −log_10_P values are shown along the left y-axis, and correspond to the variants depicted in the plot. The right y-axis shows calculated recombination rates at the chromosomal location, plotted as a solid red line. *PITX2* is located 177 kb upstream of rs2129979.

In the meta-analysis of Iceland and the Netherlands, we found a single genome-wide significant signal at 4q25 (Figs 1 and 2). The signal is represented by the common intergenic variant rs2129979 [G] (MAF_ICE_ = 29.29%; MAF_NL_ = 27.66%) associating with an increased risk of appendicitis (OR = 1.15; 95% CI = 1.10, 1.20; P = 1.8 × 10^−11^) (Table 1). Three markers show high correlation with rs2129979 (r^2^ > 0.93) in Iceland and in the main 1000Genome populations^10^. The variant is the most significant marker in the Icelandic dataset (OR = 1.14; 95% CI = 1.09, 1.19; P = 3.5 × 10^−9^), and its effect in Holland is comparable to the one in Iceland (OR = 1.19; 95% CI = 1.07, 1.32; P = 1.1 × 10^−3^; P_het_ = 0.50). In addition, we called microsatellites and structural variants from the WGS set, but found no such markers that are highly correlated with or more significant than rs2129979. After conditional analysis, no further variants associated with appendicitis.

**Table 1:** Association of rs2129979 and its three fully correlated variants with appendicitis in Iceland and the Netherlands. The minor and major alleles are the same in Iceland and the Netherlands, and all effects are presented for the minor allele. A chi-square test was used to compute *P*-values.

Marker^*^	Position	Allele (min/maj)	Iceland (7,427 cases, 340,069 controls)			The Netherlands (1,139 cases, 4,587 controls)			Combined (8,566 cases, 344,656 controls)		Relation to rs2129979	
			MAF (%)	OR (95% CI)	P	MAF (%)	OR (95% CI)	P	OR (95% CI)	P	Distance (bp)	r^2^_ICE_
rs2129979	chr4:110799841	G/T	29.3	1.14 (1.09, 1.19)	3.5 × 10^−9^	27.7	1.19 (1.07, 1.32)	1.1 × 10^−3^	1.15 (1.10, 1.20)	1.8 × 10^−11^	—	—
rs11931959	chr4:110798529	G/A	29.3	1.14 (1.09, 1.19)	3.9 × 10^−9^	27.7	1.19 (1.07, 1.32)	1.0 × 10^−3^	1.15 (1.10, 1.20)	2.1 × 10^−11^	1,589	1.00
rs2171591	chr4:110798252	A/G	29.3	1.14 (1.09, 1.19)	4.0 × 10^−9^	27.7	1.19 (1.07, 1.32)	1.1 × 10^−3^	1.15 (1.10, 1.20)	2.1 × 10^−11^	1,459	1.00
rs17042195	chr4:110798382	C/G	29.3	1.14 (1.09, 1.19)	4.1 × 10^−9^	27.7	1.19 (1.07, 1.32)	1.1 × 10^−3^	1.15 (1.10, 1.20)	2.2 × 10^−11^	1,312	1.00

^*^Imputation information = 1.00;

MAF = minor allele frequency; OR = odds ratio; CI = confidence interval.

We assessed the genotypic effect of rs2129979 in Iceland under a full model limited to chip-typed individuals in Iceland (n = 151,677; Supplementary Table 4). The risk of appendicitis is significantly greater for homozygous carriers of the minor allele than for heterozygotes (P = 7.0 × 10^−4^). The genotypic effects of rs2129979 on appendicitis are consistent with an additive mode of inheritance.

**Figure 3:**
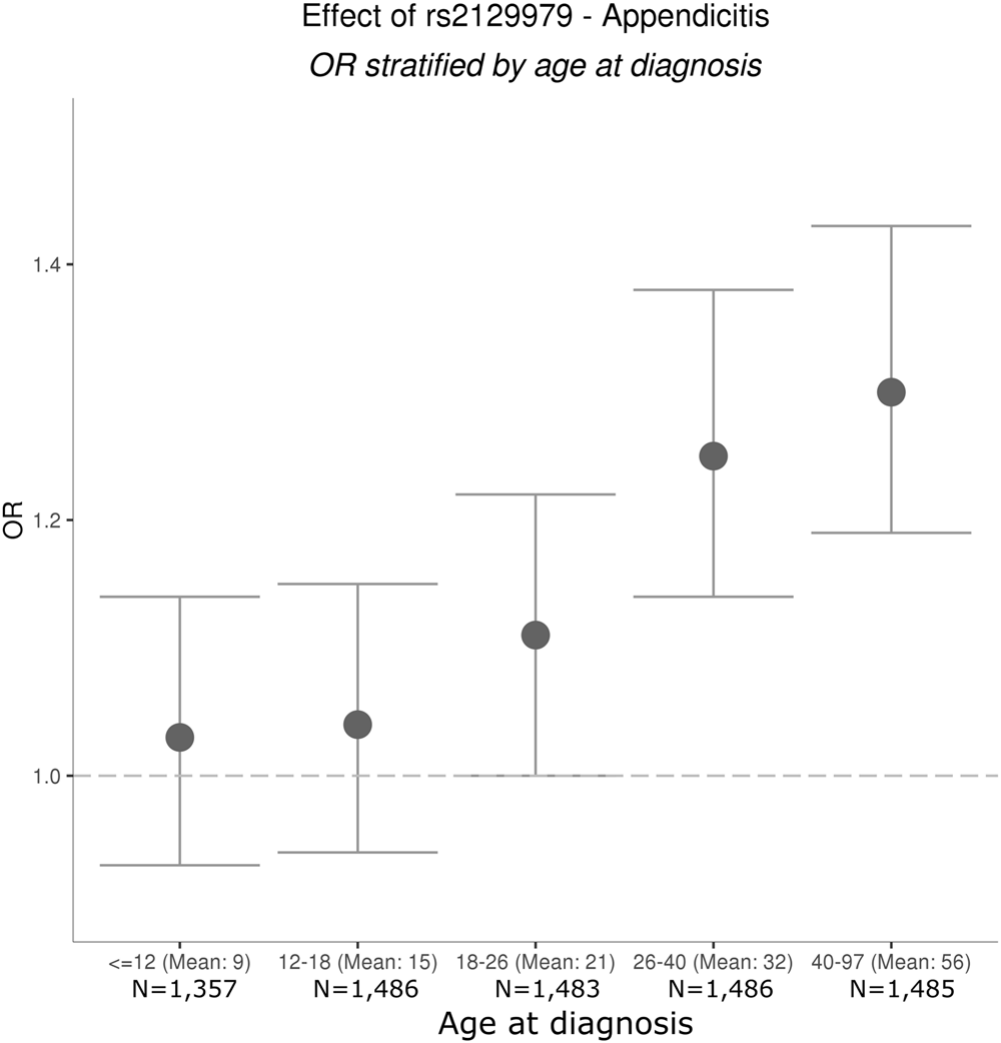
Effect of rs2129979 on appendicitis risk in Iceland. Age quintiles are shown on the x-axis, and odds ratio (OR) on the y-axis. The cases (N = 7,297) were stratified by age at diagnosis and logistic regression was performed per quintile with the same group of controls (N = 307,292), adjusting for sex and county. Each grey dot represents the OR of rs2129979 for individuals in the age group in question, and the error bars represent the 95% confidence intervals of the OR.

We found that rs2129979[G] had a significant and positive correlation with age of appendicitis diagnosis among cases in Iceland (P = 3.9 × 10^−5^), but a significant association was not seen in the Dutch cohort (P = 0.43). The combined effect on age of diagnosis for both datasets is about two years later per copy of the rs2129979 G allele (P = 7.2 × 10^−4^). The median age of diagnosis for all cases in Iceland based on hospital record dates was 22 years, consistent with published data from the US^1^. When we divide Icelandic appendicitis patients into age-at-diagnosis quintiles, the appendicitis risk conferred by rs2129979 increased monotonically with age (from 1.03 to 1.30), and was only significant for the 3 strata corresponding to an adult age (Supplementary Tables 1 and 5, Fig. 3). When we stratified our case control test in Iceland for cases above or below the median age of diagnosis, no other signals besides rs2129979 were detected (Supplementary Figures 1 and 2).

No significant differences in variant frequency were found when the Icelandic appendicitis group was subdivided by sex, or presence of peritonitis (data not shown). We found no association of rs2129979 with any of 16 other phenotypes related to diseases of the colorectum, or infectious and inflammatory processes (Supplementary Tables 6 and 7).

### Functional Annotation

The closest protein-coding gene to rs2129979 and its correlates is the homeobox transcription factor *PITX2*, located around 170 kb upstream from the variant. We examined eQTL data from adipose tissue and blood. We did not find rs2129979 to affect *PITX2* expression in adipose tissue (P = 0.66; N = 684), and expression was too low in blood for an analysis to be conducted (N = 2,512). No variants affecting expression of *PITX2* are reported by GTEx Portal^11^. Furthermore, rs2129979 did not affect expression of other nearby genes in our data or in GTEx.

The three variants are strongly correlated (r^2^ ≥ 0.93) with rs2129979 in the four main 1000Genome super-populations (AFR, AMR, EUR, ASN)^10^ (Supplementary Table 8), as well as in Iceland (r^2^ = 1.00) (Table 1). The four markers are all located within a 1.6 kb region. All four markers are reported to be within gastrointestinal enhancer histone marks specific to the fetal small intestine, and the three markers correlated to rs2129979 are also within enhancers specific to the fetal large intestine^10^.

Long range expression regulation has been found to occur to a large extent within so called topologically associated domains (TAD)^12^. rs2129979 and the three correlated appendicitis risk variants overlap a 1.5 Mb long TAD (chr4:110579395-112059395), within which *PITX2* is the only protein-coding gene.

### Relationship of appendicitis and other reported signals at 4q25

**Figure 4:**
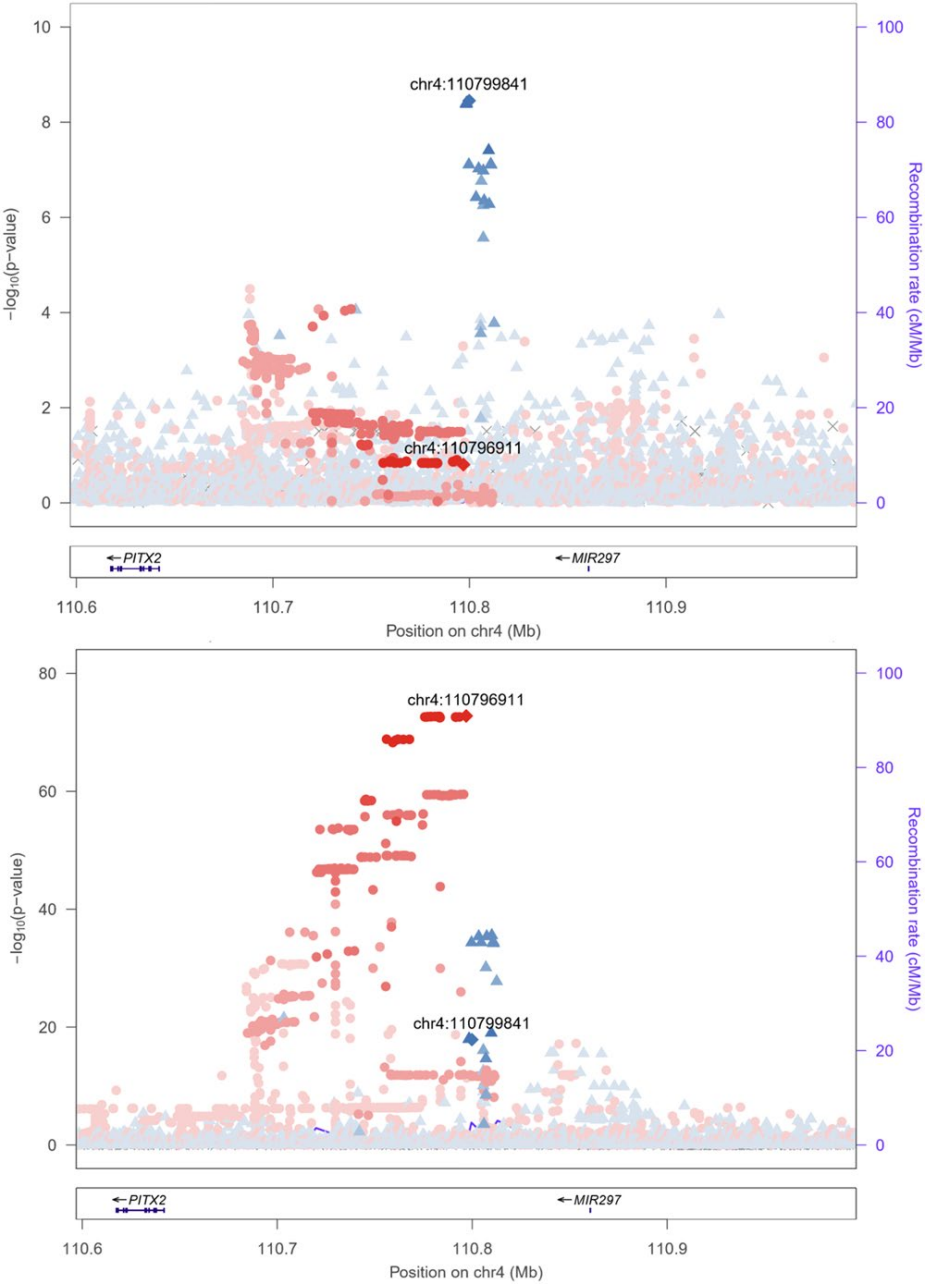
The associations of both rs2129979 (blue diamond) and rs6843082 (red diamond), and their correlated variants at 4q25, with appendicitis (above; N = 7,427) and atrial fibrillation (below; N = 13,471) in Iceland. Variants correlated to the leading variant appear in the same colour, with the degree of correlation represented by the colour saturation. Individual locus plots for the two phenotypes are shown in Supplementary Figures 6 and 7.

Two sequence variants (rs2200733 [T] and rs10033464 [T]) at the 4q25 locus have been reported to associate with increased risk of atrial fibrillation (AF) in the Icelandic population^13^ (Table 2). rs2200733 correlates modestly with rs2129979 (r^2^ = 0.32), and weakly with rs10033464 (r^2^ = 0.04). Although the markers are physically close, the association signals are fully distinct (Fig. 4, Supplementary Figure 3), suggesting two separate biological pathways. Two further AF signals have been reported in the region in studies of other populations: rs6843082 [G], and rs1448817 [G]^14^. The two variants are modestly correlated with rs2129979 in the Icelandic population (r^2^ = 0.11–0.38; Table 2). The distance between the reported AF markers and the signal represented by rs2129979 ranges from 236 bp to 80 kb. None of the four variants reported to associate with AF associate with risk of appendicitis in the Icelandic dataset after adjusting for the rs2129979 signal (all P > 1 × 10^−3^).

**Table 2:** Association of the appendicitis and previously reported atrial fibrillation signals at 4q25 with appendicitis (APP; N = 7,427) and atrial fibrillation (AF; N = 13,471) in Iceland. A chi-square test was used to compute *P*-values.

Publication PMID	Marker	Position	Allele (min/maj)	MAF (%)	OR_AF_ (95% CI)	P_AF_	OR_APP_ (95% CI)	P_APP_	Distance (BP)	r^2^ with rs2129979 (IS)	D′
Current	rs2129979	chr4:110799841	G/T	29.3	1.17 (1.13, 1.21)	1.3 × 10^−18^	1.14 (1.09, 1.19)	3.5 × 10^−9^	NA	NA	NA
17603472	rs2200733	chr4:110789013	T/C	11.8	1.49 (1.42, 1.56)	3.0 × 10^−60^	1.07 (1.01, 1.14)	0.03	10,828	0.32	1
17603472	rs10033464	chr4:110799605	T/G	8.32	1.23 (1.16, 1.30)	2.0 × 10^−12^	0.99 (0.93, 1.05)	0.74	236	0.04	1
26497660	rs6843082	chr4:110796911	G/A	20.1	1.43 (1.38, 1.49)	1.6 × 10^−73^	1.04 (0.98, 1.30)	0.16	2,930	0.11	0.42
26497660	rs1448817	chr4:110719897	G/A	24.8	1.25 (1.20, 1.30)	1.3 × 10^−32^	1.09 (1.04, 1.14)	2.0 × 10^−4^	79,944	0.38	0.69

MAF = minor allele frequency; OR = odds ratio; CI = confidence interval.

## Discussion

We detected a significant association between four correlated sequence variants at 4q25 and appendicitis. The closest protein-coding gene is *PITX2*, encoding the transcription factor pituitary homeobox 2. The region containing *PITX2* has previously been described in the context of atrial fibrillation^13, 15^, and several papers have provided speculation as to the direct functional involvement of *PITX2* ^16, 17^. *PITX2* has, in addition, been conclusively demonstrated to be important in the determination of right-left symmetry in development (i.e. situs-specific morphogenesis)^18^. Mutations in *PITX2* are the cause of several Mendelian diseases including Rieger syndrome, a morphogenesis disorder that can present with systemic anomalies that include imperforate anus and anal stenosis^19^. Many individuals with situs anomalies also display various intra-abdominal and/or gastrointestinal abnormalities^20^. *PITX2* signaling has also been shown to regulate the development of the cecum in mice, an intestinal structure directly connected to the appendix^21, 22^, and to regulate gut looping and vascular development in the gut of various animal models^23, 24^.

The small intestine and colon are among the tissues in which *PITX2* is expressed the most^25^. In the Roadmap data^26^, rs2129979 and its three correlates are assigned an enhancer state specifically in fetal small intestine, and all four overlap a H3K4me1 enhancer histone mark region in intestinal cell lines. Two of the variants are predicted^27^ to alter binding of transcription factors with tissue-specific expression in colon and/or small intestine: rs2129979 disrupts a binding site for EVI1 (encoded by the gene *MECOM*), and rs17042195 introduces an Early B-cell Factor (EBF) binding motif. This collection of evidence suggests that these variants affect appendicitis risk through modulation of tissue specific regulation of the *PITX2* gene in intestinal tissues, and in a time-dependent fashion. We note that the second-closest protein-coding gene is *ENPEP*, but no clear links can be drawn between it and the effects of variants at 4q25 based on the literature.

It is unclear if rs2129979 or correlated variants at 4q25 directly increase the risk of appendicitis, the likelihood of being diagnosed with the condition, or patient prognosis and risk of complications. While the relationship between situs anomalies and appendicitis are unclear, we cannot exclude that an anatomical abnormality could affect the likelihood of appendicitis diagnosis.

The association of rs2129979 with appendicitis is limited to patients diagnosed above the median age of diagnosis. We note that the incidence of appendicitis is much lower among the adult population where we observe a higher risk conferred by rs2129979.

Our data do not indicate a direct relationship between the signals for atrial fibrillation and appendicitis at 4q25, although the reported appendicitis signal is close to previously reported atrial fibrillation signals. We speculate that both signals are encompassed within an important hub for tissue-specific regulatory elements close to 4q25.

We have discovered an association between a common sequence variant and risk of appendicitis at *PITX2*. We only detected association for cases diagnosed during adulthood, suggesting different pathogenesis for the disease in different age groups. Further studies are required, however, to elucidate biological mechanisms underlying this association.

## Methods

### Study subjects from Iceland

This study is based on whole-genome sequence data from the whole blood of 15,220 Icelanders participating in various disease projects at deCODE genetics. In addition, 151,677 Icelanders have been genotyped using Illumina SNP chips and genotype probabilities for untyped relatives has been calculated based on Icelandic genealogy.

All participating individuals who donated blood, or their guardians, provided written informed consent. The family history of participants donating blood was incorporated into the study by including the phenotypes of first- and second-degree relatives and integrating over their possible genotypes.

All sample identifiers were encrypted in accordance with the regulations of the Icelandic Data Protection Authority. Approval for the study was provided by the National Bioethics Committee (ref:VSNb2015100030/03.03). Personal identities of the participants and biological samples were encrypted by a third-party system approved and monitored by the Icelandic Data Protection Authority. The National Bioethics Committee approved the study, including the protocol, methodology and all documents presented to the participants, and all methods were performed in accordance with the relevant guidelines and regulations.

To identify appendicitis cases, we searched for patients with International Classification of Diseases (ICD10) codes, diagnosis codes K35-K38, indicative of appendicitis at Landspitali—The National University Hospital of Iceland in Reykjavik (LUH), a community hospital for half of Iceland’s population. The records spanned from 1983–2015. A total of 7,267 appendicitis cases were included in the association analysis; 175 of these were whole-genome sequenced, 3,372 were genotyped using various Illumina chips and imputed using long-range phased haplotypes, and genotype probabilities for 3,720 were imputed on the basis of information from genotyped close relatives. Controls comprised individuals recruited through different genetic research projects at deCODE. Individuals in the appendicitis cohort were excluded from the control group. Of the controls, 7,534 were whole-genome sequened, 134,153 were genotyped by chip, and 185,447 were imputed on the basis of the genotypes of close relatives. The total number of controls was 327,134.

The process used to whole-genome sequence the Icelandic population, and the subsequent imputation from which the data for this analysis were generated has been extensively described in a recent publication^28^.

### Study subjects from the Netherlands

The Dutch cases consisted of 1,139 individuals with self-reported appendicitis and/or appendectomy. Of those, 659 cases were recruited in a project entitled “Nijmegen Biomedical Study”^29^. The remaining cases were recruited through previously described studies on bladder cancer (225 cases)^30^, prostate cancer (109 cases)^31^, breast cancer (109 cases)^32^ and ovarian cancer (37 cases)^33^. The 4,587 Dutch controls were individuals from the Nijmegen Biomedical Study that did not report having had appendicitis or appendectomy.

The study protocols of all the cancer studies and the Nijmegen Biomedical Study were approved by the Institutional Review Board of the Radboud University Medical Center and all study subjects gave written informed consent.

The Dutch study samples were genotyped using Omni-1 Quad-bead chips (Illumina, San Diego, CA, USA). Variants were excluded if they (i) had <94% yield, (ii) had <1% MAF, (iii) failed Hardy-Weinberg test (P < 1 × 10^−6^) or (iv) showed significant (P < 1 × 10^−6^) difference between genotype batches. Samples with <94% yield were excluded. The resulting genotypes were phased using SHAPEIT (v2.790)^34^, and used to impute un-genotyped variants using IMPUTE2 (v2.3.2)^35^. The Dutch study samples were imputed using The Genome of the Netherlands^36^ (GoNL) dataset generated by whole-genome sequencing of 249 Dutch trios (498 unrelated parents and 249 children).

#### Population structure

To study the population structure and the ancestry of samples in the Dutch cohort we used the ADMIXTURE (v 1.2)^37^ and EIGENSOFT (v 6.0.1)^38^ software. Samples were excluded if they were identified as ethnic outliers and to adjust for remaining population substructure 10 principle components were included as covariates in the subsequent association analysis.

#### Association testing

Logistic regression was used to test for association between variants and disease, assuming a multiplicative model, treating disease status as the response and expected genotype counts from imputation as covariates. For the Icelandic cohort this was done using software developed at deCODE genetics^28^, but the Dutch cohort was analyzed using the SNPTEST (v.2.5) software^39^. Testing was performed using the likelihood ratio statistic.

#### Meta-analysis

Variants in the GoNL imputation datasets were mapped to NCBI Build38 positions and matched to the variants in the Icelandic dataset based on allele variation. Results from the different study groups were combined using a Mantel-Haenszel model^40^ in which the groups were allowed to have different population frequencies for alleles and genotypes but were assumed to have a common OR. Heterogeneity was tested by comparing the null hypothesis of the effect being the same in all populations to the alternative hypothesis of each population having a different effect using a likelihood ratio test. *I* ^2^ lies between 0% and 100% and describes the proportion of total variation in study estimates that is due to heterogeneity.

## Electronic supplementary material

Supplementary Tables and Figures
