# Supplementary material for: Sequence variant at 4q25 near PITX2 associates with appendicitis
Source: Sci Rep. 2017 Jun 8;7:3119. doi: 10.1038/s41598-017-03353-0 (PMC5465083; doi:10.1038/s41598-017-03353-0)
Supplement: Supplementary file 1 — Supplementary Tables and Figures [file 41598_2017_3353_MOESM1_ESM.doc]

**Sequence variant at 4q25 near *PITX2* associates with appendicitis**

Ragnar P Kristjansson^1,*^, Stefania Benonisdottir^1,*^, Asmundur Oddsson^1^, Tessel E Galesloot^2^, Gudmar Thorleifsson^1^, Katja K Aben^2,3^, Olafur B Davidsson^1^, Stefan Jonsson^1^, Gudny A Arnadottir^1^, Brynjar O Jensson^1^, G. Bragi Walters^1^, Jon K Sigurdsson^1^, Snaevar Sigurdsson^1^, Hilma Holm^1^, David O Arnar^4^, Gudmundur Thorgeirsson^1,4,5^, Kristin Alexiusdottir^4^, Ingileif Jonsdottir^1,5^, Unnur Thorsteinsdottir^1,5^, Lambertus A Kiemeney^2^, Thorvaldur Jonsson^5,6^, Daniel F Gudbjartsson^1,7^, Thorunn Rafnar^1^, Patrick Sulem^1^, Kari Stefansson^1,5^

1. deCODE genetics/Amgen, Inc., Reykjavik 101, Iceland

2. Radboud University Medical Center, Radboud Institute for Health Sciences, Department for Health Evidence, PO Box 9101, 6500 HB Nijmegen, The Netherlands

3. Netherlands Comprehensive Cancer Organisation. PO Box 19079, 3501 DB Utrecht, The Netherlands

4. Department of Medicine, Landspítali – The National University Hospital of Iceland, Hringbraut, 101 Reykjavik, Iceland

5. Faculty of Medicine, University of Iceland, Reykjavik, Iceland

6. Department of Surgery, Landspítali – The National University Hospital of Iceland, Hringbraut, 101 Reykjavik, Iceland

7. School of Engineering and Natural Sciences, University of Iceland, Reykjavik, Iceland.

* These authors contributed equally to this work

**Supplementary Tables and Figures**

**Supplementary Table 1:** Association of rs2129979 with appendicitis in cases subdivided into age quintiles. A chi-square test was used to compute P-values.

| **Age group** | **N cases** | **N controls** | **P** | **OR** | **95% CI** |
| --- | --- | --- | --- | --- | --- |
| ≤12 | 1,357 | 307,292 | 0.59 | 1.03 | 0.93, 1.14 |
| 12-18 | 1,486 | 307,292 | 0.46 | 1.04 | 0.94, 1.15 |
| 18-26 | 1,483 | 307,292 | 0.04 | 1.11 | 1.00, 1.22 |
| 26-40 | 1,486 | 307,292 | 3.9x10^-6^ | 1.25 | 1.14, 1.38 |
| 40-97 | 1,485 | 307,292 | 4.3x10^-8^ | 1.30 | 1.19, 1.43 |

OR = odds ratio; CI = confidence interval

**Supplementary Table 2:** Estimated appendicitis risk ratio for mates and close relatives in Iceland based on cross-matching appendicitis cases with genealogy.

| **Relation** | **RR** | **95% CI** | **P_RR_** | **N (affected relative)** |
| --- | --- | --- | --- | --- |
| **Mates** | 1.34 | 1.08, 1.64 | 0.02 | 192 |
| **Parents** | 1.80 | 1.63, 1.99 | < 1.10^-5^ | 377 |
| **Father** | 1.72 | 1.47, 1.99 | < 1.10^-5^ | 170 |
| **Mother** | 1.88 | 1.64, 2.15 | < 1.10^-5^ | 207 |
| **Children** | 1.66 | 1.50, 1.85 | < 1.10^-5^ | 377 |
| **Siblings** | 1.95 | 1.79, 2.11 | < 1.10^-5^ | 1,106 |
| **Grandparents** | 1.55 | 1.34, 1.79 | < 1.10^-5^ | 207 |
| **Cousins** | 1.26 | 1.21, 1.32 | < 1.10^-5^ | 4,144 |

RR = Relative risk; CI = confidence interval

**Supplementary Table 3:** The ICD-10 codes used to identify appendicitis cases in Iceland (N=8,160). Diagnoses reported using ICD-9 were converted to ICD-10.

|  | **ICD Code** | **Description** | **N cases** |
| --- | --- | --- | --- |
| **K35** | K35.0^†^ | Acute appendicitis with generalized peritonitis | 514 |
|  | K35.1^†^ | Acute appendicitis with peritoneal abscess | 168 |
|  | K35.2^‡^ | Acute appendicitis with generalized peritonitis | 474 |
|  | K35.3^‡^ | Acute appendicitis with localized peritonitis | 835 |
|  | K35.8^‡^ | Acute appendicitis, other and unspecified | 2,579 |
|  | K35.9^†^ | Acute appendicitis, unspecified | 2,995 |
| **K36** | K36 | Other appendicitis | 64 |
| **K37** | K37 | Unspecified appendicitis | 205 |
| **K38** | K38.0 | Hyperplasia of appendix | 0 |
|  | K38.1 | Appendicular concretions | 3 |
|  | K38.2 | Diverticulum of appendix | 2 |
|  | K38.3 | Fistula of appendix | 0 |
|  | K38.8 | Other specified diseases of appendix | 13 |
|  | K38.9 | Disease of appendix, unspecified | 8 |

† K35 subcategories used prior to 2010 ICD-10 revision; ‡ K35 subcategories used after 2010 ICD-10 revision

**Supplementary Table 4: Genotypic effect of rs2129979; G is the minor allele and T is the major allele. Logistic regression was performed, and county, gender, and year of birth were used as additional covariates. The effect of rs2129979 on appendicitis is consistent with an additive mode of inheritance. The analysis was limited to chip-typed individuals. Frequency of genotypes: [TT] = 49.94%, [GT] 41.44%, [GG] 8.61%. A chi-square test was used to compute P-values.**

|  | **Genotype** | | |
| --- | --- | --- | --- |
|  | **GG vs. TT** | **GT vs. TT** | **GG vs. GT** |
| **Appendicitis cases** | 1,919 | 2,964 | 1,761 |
| **Appendicitis controls** | 77,724 | 120,695 | 65,811 |
| **OR (95% CI)** | 1.33 (1.18, 1.49) | 1.09 (1.01, 1.18) | 1.23 (1.09, 1.39) |
| **P** | 7.4x10^-6^ | 3.5x10^-2^ | 1.4x10^-3^ |

OR = odds ratio; CI = confidence interval

**Supplementary Table 5:** GWAS results for rs2129979 and appendicitis sub-phenotypes in Iceland. Age information was unavailable for 17 patients. A chi-square test was used to compute P-values.

| **Phenotype** | **N** | **OR** | **95% CI** | **P** |
| --- | --- | --- | --- | --- |
| Appendicitis | 7,427 | 1.14 | 1.09, 1.20 | 3.5x10^-9^ |
| Appendicitis diagnosed at age 22 or younger vs. control | 3,959 | 1.04 | 0.98, 1.11 | 0.22 |
| Appendicitis diagnosed after age 22 vs. control | 3,451 | 1.23 | 1.16, 1.30 | 3.1x10^-12^ |
| Appendicitis before age 22 vs. after age 22 | 7,410 | 0.85 | 0.75, 0.97 | 1.7x10^-2^ |

OR = odds ratio; CI = confidence interval

***Supplementary Table 6:*** *Association of rs2129979 with colorectal, infectious, and inflammatory diseases in Iceland. A chi-square test was used to compute P-values.*

| **Phenotype** | **Cases (n)** | **OR (95% CI)** | **Pval** |
| --- | --- | --- | --- |
| Asthma | 13,190 | 1.03 (0.99, 1.07) | 0.17 |
| Diverticular Disease | 5,425 | 0.97 (0.92, 1.02) | 0.23 |
| Ulcerative Colitis | 1,596 | 1.05 (0.97, 0.14) | 0.23 |
| Inflammatory Bowel Disease | 2,079 | 1.05 (0.97, 1.14) | 0.24 |
| Crohn’s Disease | 399 | 1.03 (0.88, 1.20) | 0.71 |
| Colorectal Cancer | 3,987 | 0.99 (0.93, 1.06) | 0.77 |
| Bacterial Invasive Disease | 3,266 | 0.99 (0.90, 1.09) | 0.84 |
| Tuberculosis | 8,924 | 1.00 (1.00, 1.00) | 0.96 |
| COPD | 7,357 | 1.00 (1.00, 1.00) | 0.97 |

OR = odds ratio; CI = confidence interval

**Supplementary Table 7:** Association of rs2129979 with white blood cell and neutrophil counts in Iceland. A chi-square test was used to compute P-values.

| **Phenotype** | **Individuals (n)** | **Effect** | **Pval** |
| --- | --- | --- | --- |
| White blood cell count | 273,110 | 0.00 | 0.95 |
| Neutrophils count | 252,010 | 0.00 | 0.96 |

**Supplementary Table 8:** Frequencies of rs2129979 and the three highly correlated variants in the four major 1000Genomes Populations, and the relative conservation status of each position as defined by Genomic Evolutionary Rate Profiling (GERP). The most conserved of the four is rs2129979; a GERP score of 3.72 makes it one of approximately top 2% most conserved non-coding variants^1^.

|  |  |  |  |  | **1000Genomes Population** | | | |  |
| --- | --- | --- | --- | --- | --- | --- | --- | --- | --- |
| **Marker** | **Position** | **Allele (min/maj)** | **MAF_IS_ (%)** | **MAF_NL_ (%)** | **AFR** | **AMR** | **ASN** | **EUR** | **GERP** |
| rs2129979 | chr4:110799841 | G/T | 29.3 | 27.7 | 0.58 | 0.45 | 0.66 | 0.30 | 3.72 |
| rs11931959 | chr4:110798529 | G/A | 29.3 | 27.7 | 0.58 | 0.45 | 0.66 | 0.30 | 1.12 |
| rs2171591 | chr4:110798252 | A/G | 29.3 | 27.7 | 0.58 | 0.44 | 0.66 | 0.30 | 1.09 |
| rs17042195 | chr4:110798382 | C/G | 29.3 | 27.7 | 0.58 | 0.45 | 0.66 | 0.30 | -0.974 |

MAF = minor allele frequency; AFR = African; AMR = Ad Mixed American; ASN = Asian; EUR = European


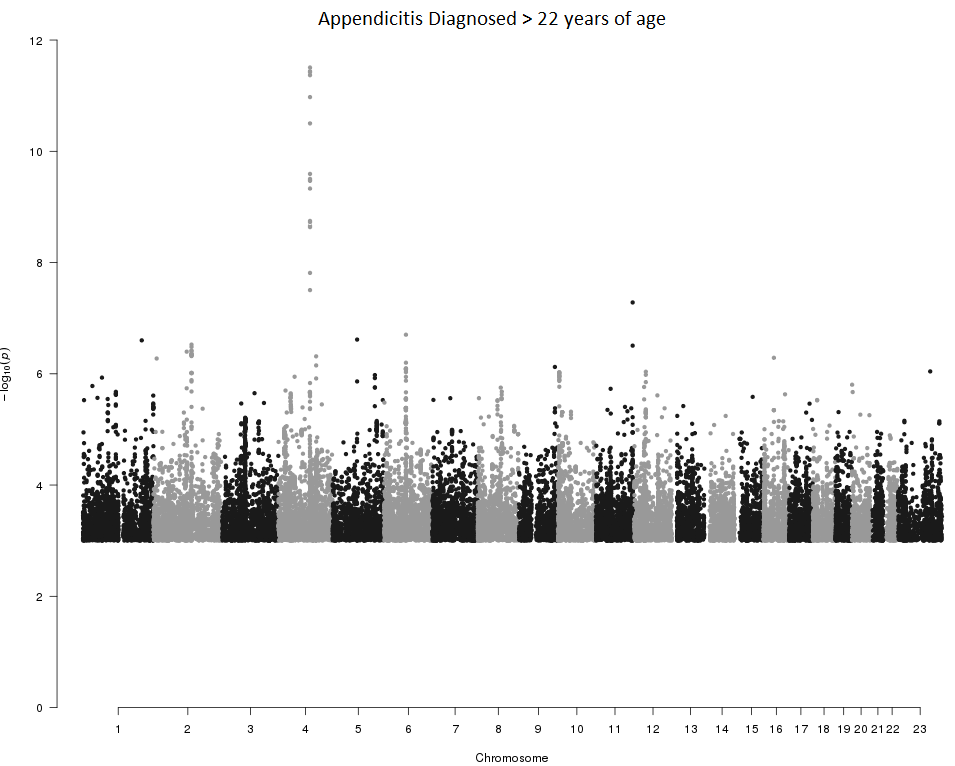


**Supplementary Figure 1:** Manhattan plot for the Icelandic appendicitis GWAS of patients diagnosed after the age of 22 (N = 3,451). 4q25 harbors a genome-wide significant signal. Variants are plotted by chromosomal position (x-axis) and log_10_P values (y-axis).


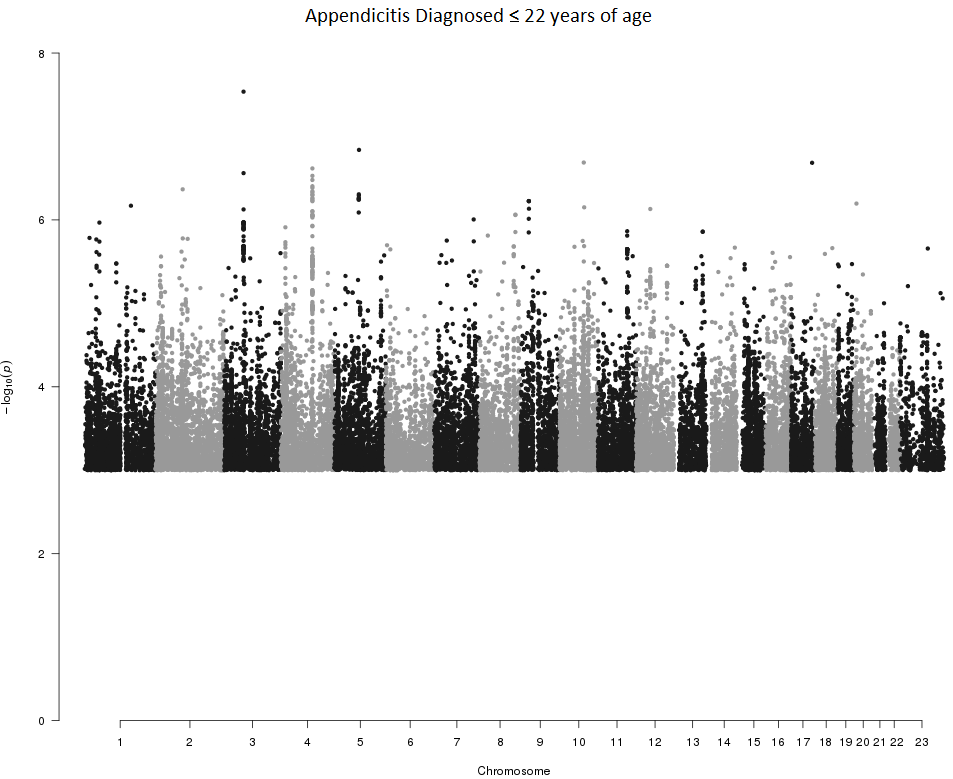


**Supplementary Figure 2:** Manhattan plot for the Icelandic appendicitis GWAS of patients diagnosed at age 22 or earlier (N = 3,959). No significant signals are observed. Variants are plotted by chromosomal position (x-axis) and log_10_P values (y-axis).


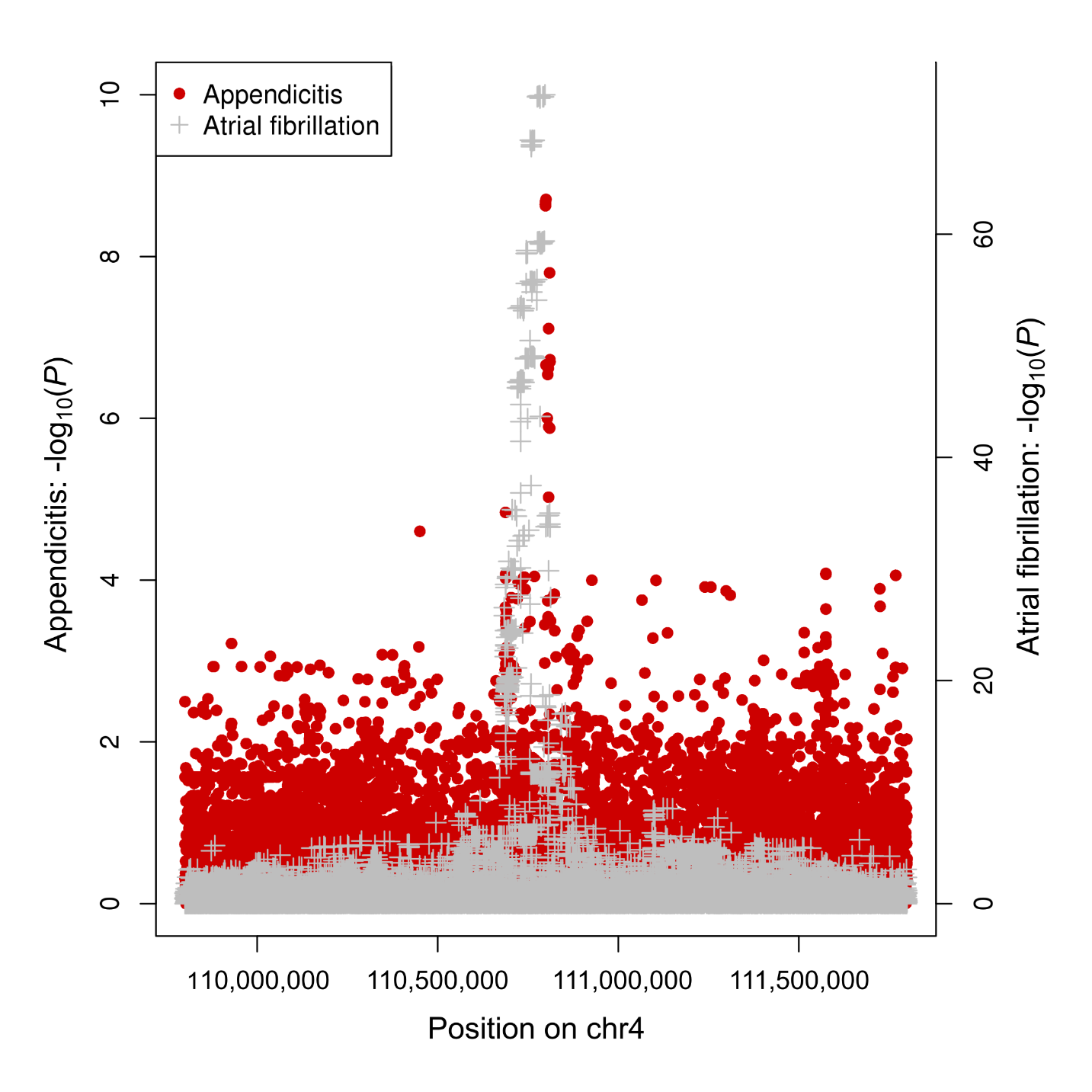


**Supplementary Figure 3:** The association signals observed at 4q25 for appendicitis (red circles; N = 7,427) and atrial fibrillation (gray crosses; N = 13,471). –log_10_P values are shown along the left y-axis, and correspond to the association of variants depicted in the plot with appendicitis. –log_10_P values corresponding to the association of variants depicted in the plot with atrial fibrillation are shown along with right y-axis. The two signals, although distinct, are physically close.


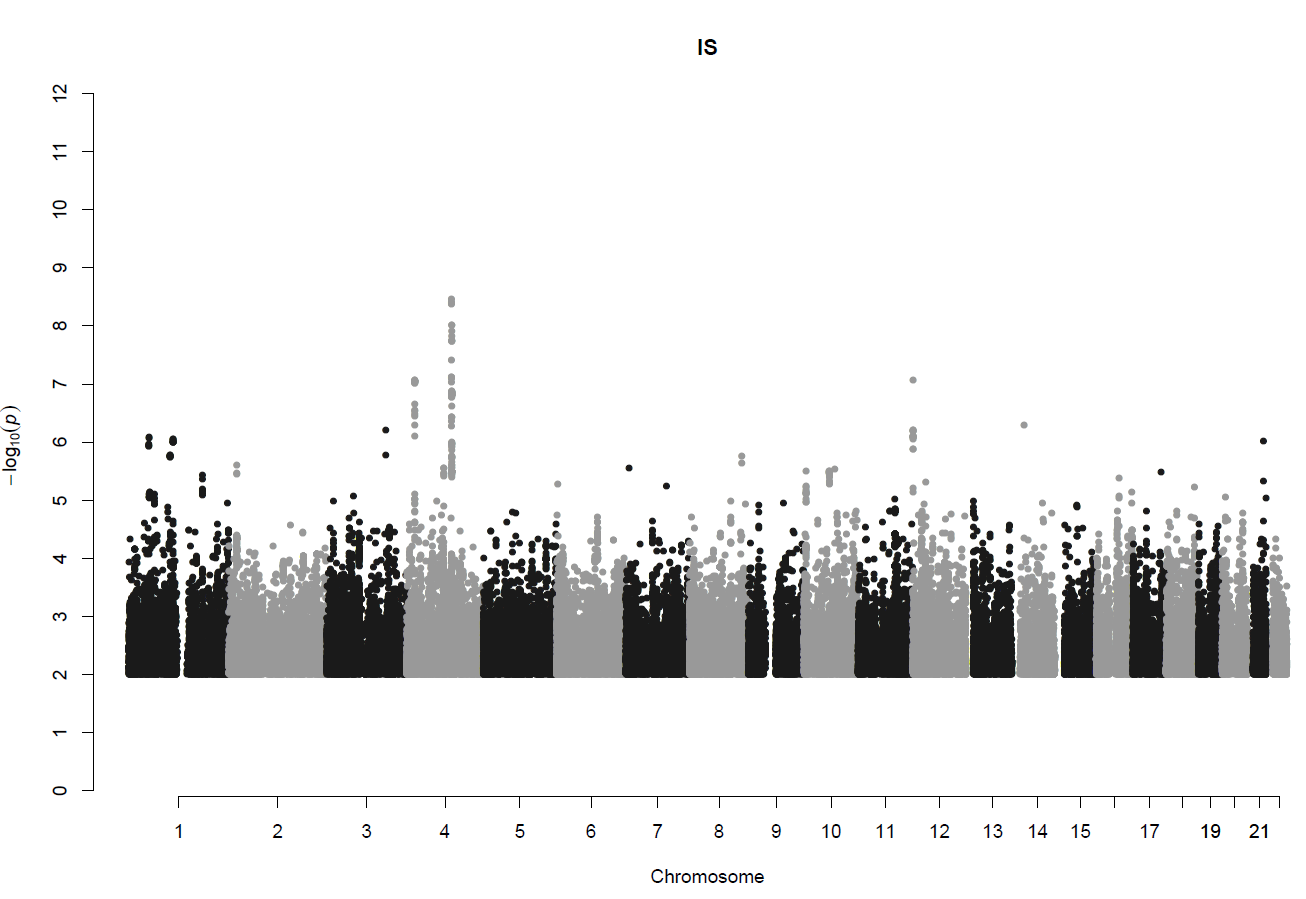


**Supplementary Figure 4:** Manhattan plot for the Icelandic appendicitis GWAS (N = 7,427). No genome-wide significant signals can be seen, although the locus at 4q25 can be seen to approach genome-wide significance. Variants are plotted by chromosomal position (x-axis) and log_10_P values (y-axis).


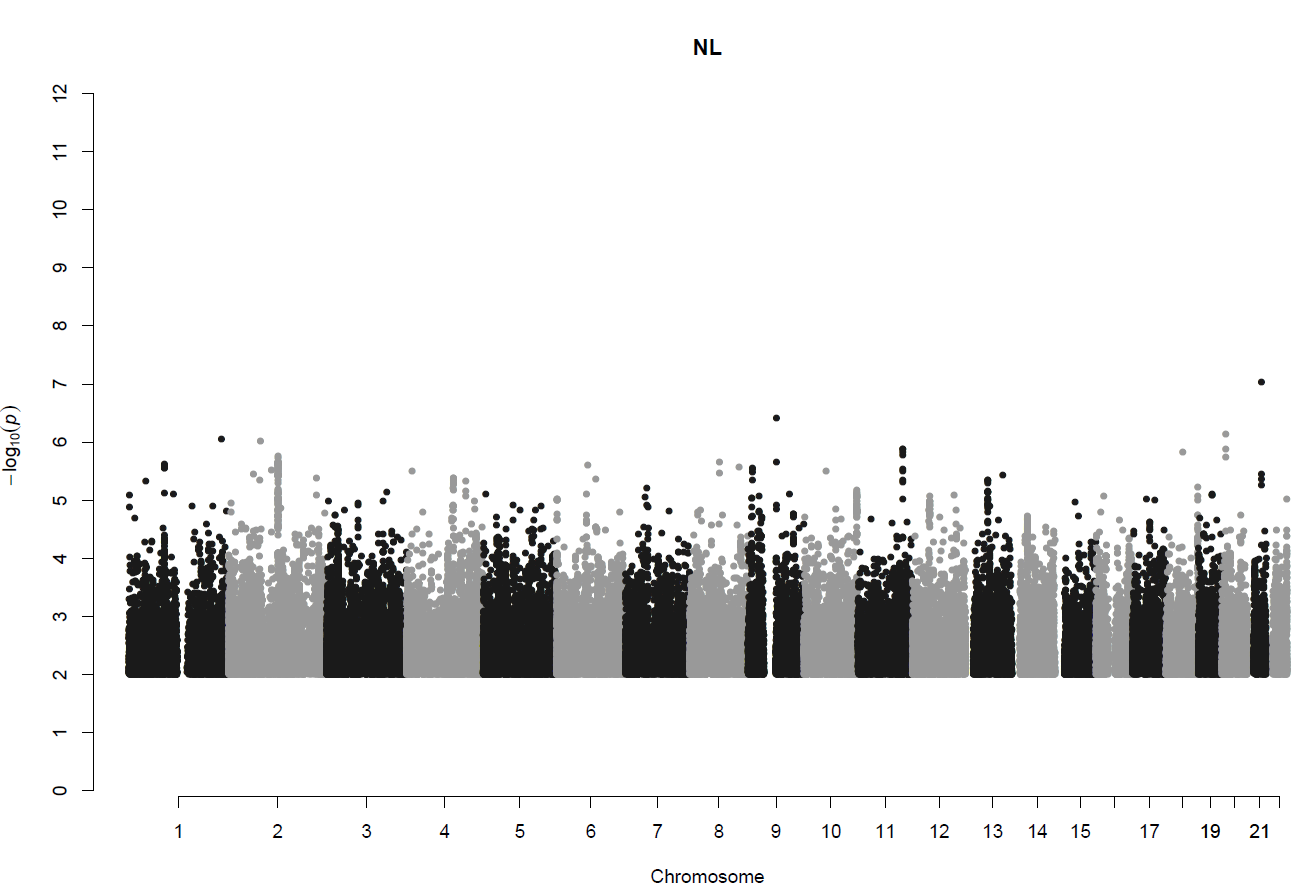


**Supplementary Figure 5:** Manhattan plot for the Dutch appendicitis GWAS (N = 1,139). No genome-wide significant signals can be seen. Variants are plotted by chromosomal position (x-axis) and log_10_P values (y-axis).


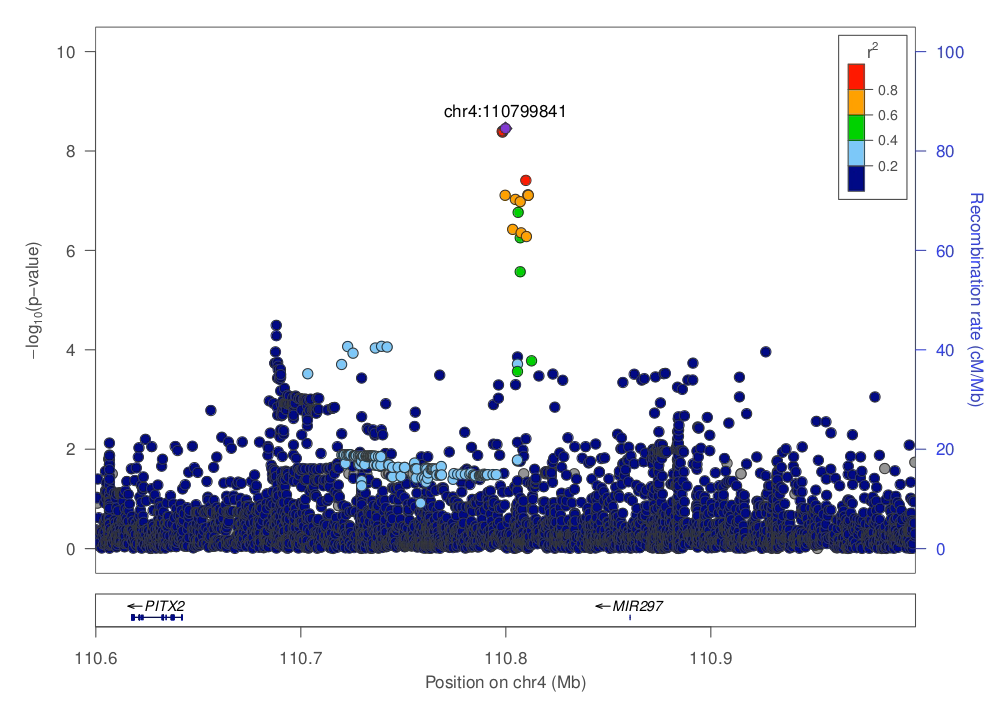


**Supplementary Figure 6:** Locus plot depicting the association of rs2129979 and correlated variants at 4q25 with appendicitis in Iceland (N = 7,427). The leading variant is shown in purple, and other variants are coloured according to correlation (r^2^) with the leading marker (legend at top-right). –log_10_P values are shown along the left y-axis, and correspond to the variants depicted in the plot. The right y-axis shows calculated recombination rates at the chromosomal location, plotted as a solid blue line. PITX2 is located 177kb upstream of rs2129979.


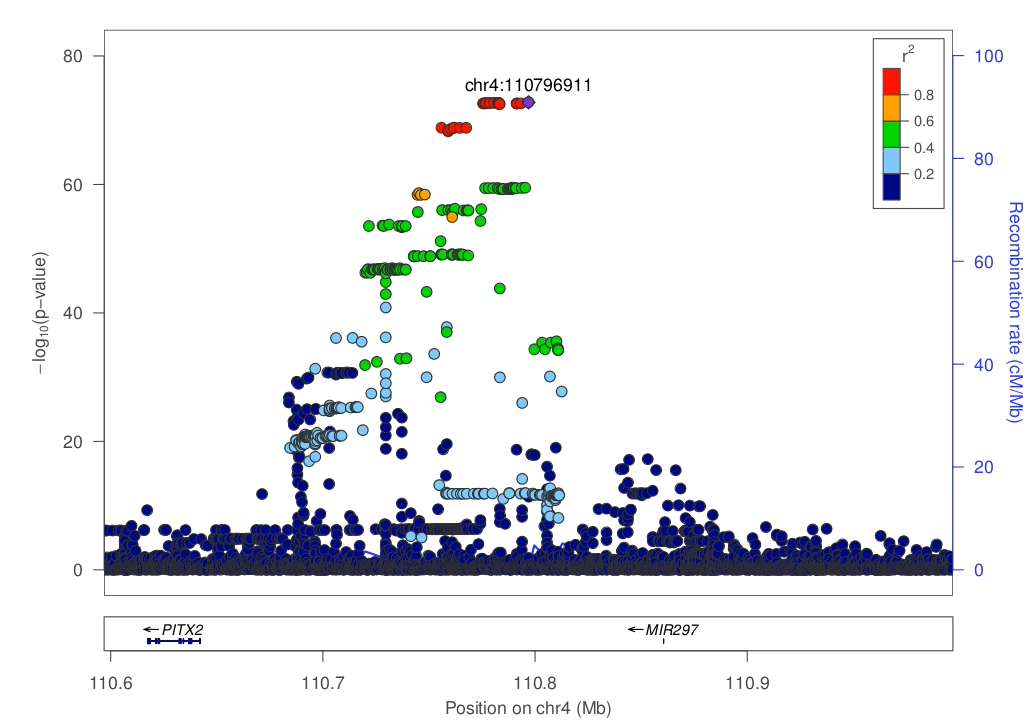


**Supplementary Figure 7:** Locus plot depicting the association of rs6843082 and correlated variants at 4q25 with atrial fibrillation in Iceland (N = 13,471). The leading variant is shown in purple, and other variants are coloured according to correlation (r^2^) with the leading marker (legend at top-right). –log_10_P values are shown along the left y-axis, and correspond to the variants depicted in the plot. The right y-axis shows calculated recombination rates at the chromosomal location, plotted as a solid blue line.


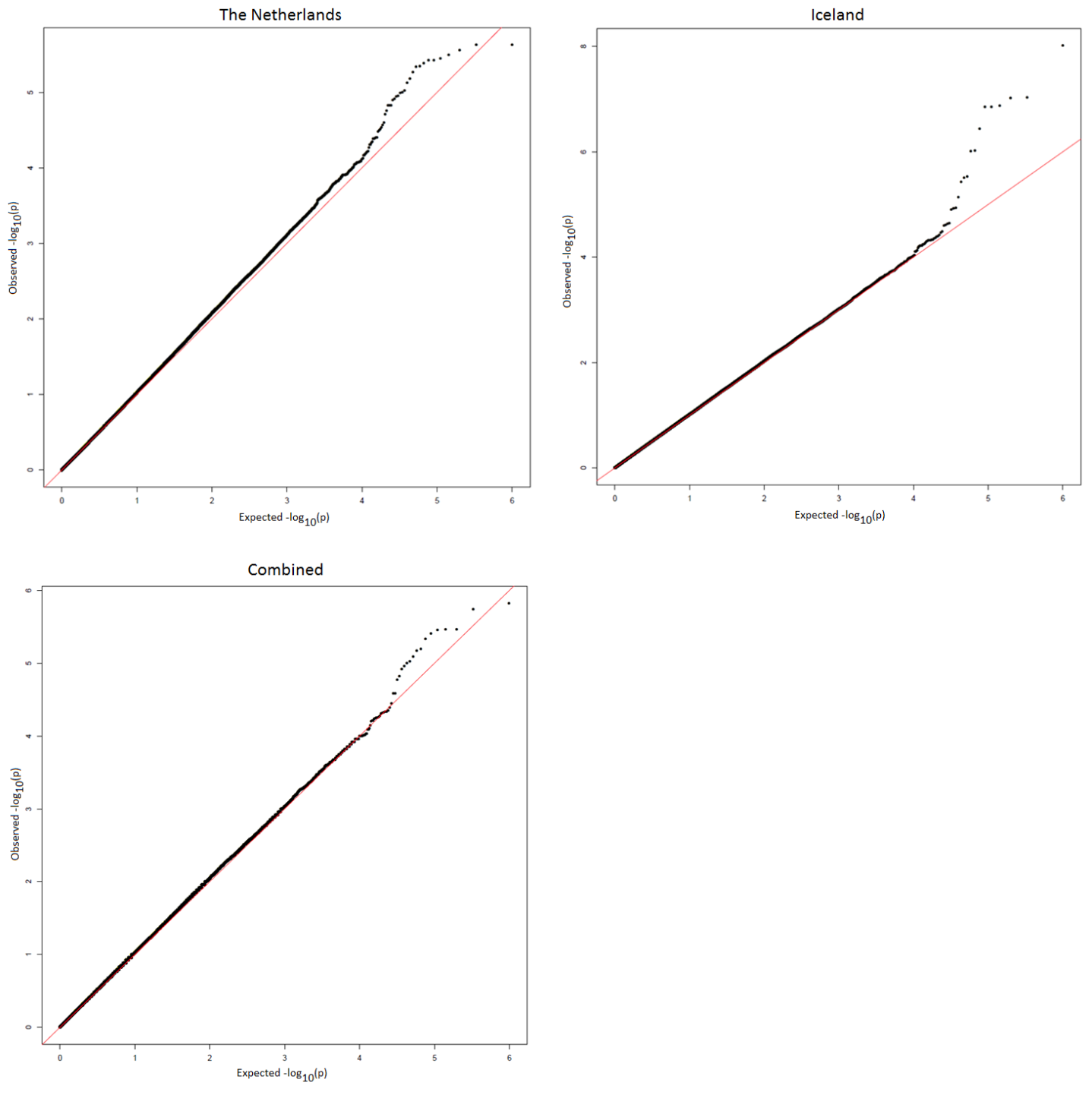


**Supplementary Figure 8:** Q-Q plots using corrected Χ^2^ statistics from the Dutch (top left; N = 1,139), Icelandic (top right; N = 7,427), and combined (bottom; N = 8,566) appendicitis GWASs.

**Supplementary References**

1. Cooper, G.M. & Shendure, J. Needles in stacks of needles: finding disease-causal variants in a wealth of genomic data*. Nat Rev Gen*e**t** 12, 628-40 (2011).
